# Determinants of acute mortality of *Hippodamia convergens* (Coleoptera: Coccinellidae) to ultra-low volume permethrin used for mosquito management

**DOI:** 10.7717/peerj.2167

**Published:** 2016-06-23

**Authors:** Robert K.D. Peterson, Collin J. Preftakes, Jennifer L. Bodin, Christopher R. Brown, Alyssa M. Piccolomini, Jerome J. Schleier

**Affiliations:** Land Resources and Environmental Sciences, Montana State University, Bozeman, Montana, United States

**Keywords:** Risk assessment, Exposure assessment, Non-target organisms, Pyrethroid, Pesticide

## Abstract

There are relatively few experimental studies and risk assessments of the effects on non-target insects from ultra-low volume (ULV) insecticides used for management of adult mosquitoes. Therefore, we evaluated factors that may influence the ability of an insect to intercept the insecticide at the time of application by using *Hippodamia convergens* (Coleoptera: Coccinellidae) in field bioassay experiments in 2011 and 2015. Treatment factors included different distances, two cage heights (ground-level and 1.5 m above ground) to the point of the application, and covered vs. uncovered cage faces (2015 only). Insecticides used included a water-based formulation (Aqua-Reslin®) and an oil-based formulation (Permanone® 30-30) of permethrin. Cage height was highly significant both years, with much less acute (i.e., short-term exposure) mortality at ground-level compared with 1.5 m. In 2011, acute mortality was less at ground-level (mean = 3.2%, median = 0%) compared to 1.5 m (mean = 85.2%, median = 100%). Cage type also was highly significant, with less mortality in covered cages compared to uncovered cages. Mortality by cage height and cage type was as follows: ground level, covered cage (mean = 2.8%, median = 0.1%); ground level, uncovered cage (mean = 41.9%, median = 9.6%); 1.5 m, covered cage (mean = 6.8%, median = 0%); 1.5 m, uncovered cage (mean = 83.7%, median = 100%). Results suggest that acute mortality to non-target insects may vary considerably based on their height and their ability to directly intercept the insecticide as the aerosol passes through the area being sprayed.

## Introduction

Within integrated pest management (IPM) programs, ultra-low volume (ULV) applications of insecticides in outdoor environments reduce populations of mosquitoes and most likely reduce the incidence of mosquito-vectored viruses in mosquitoes, non-human vertebrates, and humans, providing public health benefits to the treated areas through the prevention of disease (Carney et al., 2008; Elnaiem et al., 2008; Macedo et al., 2010; Ruktanonchai et al., 2014). ULV application technology for mosquito management is characterized by releasing into the air extremely low rates of insecticide active ingredient (e.g., 3.92 g/ha and volume of total product (e.g., 0.1 L/min) in very small droplets (e.g., 5–20 μm) to maximize aerial coverage and knock down and kill flying adult mosquitoes. With the increase in mosquito-borne pathogens such as West Nile virus (WNV), insecticides such as permethrin are being used more extensively for adult mosquito control (Davis, Peterson & Macedo, 2007; Schleier et al., 2012). Consequently, public concern about insecticides has also increased, including concern about risk to non-target insects and other arthropods (Roche, 2002; Thier, 2001).

The technology of using ULV insecticides for mosquito management was developed to maximize aerial coverage and target flying adult mosquitoes at twilight and night and not to target insects on foliage and other landscape features (Lofgren, Anthony & Mount, 1973; Mount, 1998; Mount, Biery & Haile, 1996). However, other than many years of anecdotal observations of little impact on non-target insects, there are relatively few experimental studies and risk assessments of this technology on these ecological receptors. This is especially the case for ground-applied ULV insecticides (Davis & Peterson, 2008; Schleier & Peterson, 2010b; Tietze et al., 1996). Results from the few experimental studies that have been conducted are equivocal, suggesting significant acute exposure and mortality of specific non-target insect taxa abundance in some cases but not in others (Bargar, 2012b; Boyce et al., 2007; Breidenbaugh & de Szalay, 2010; Jensen, Lawler & Dritz, 1999; Kwan et al., 2009; Tietze et al., 1996; Zhong et al., 2010; Zhong et al., 2004). Furthermore, in viewing ground-applied ULV insecticide applications as part of our previous research (Davis & Peterson, 2008; Preftakes, Schleier & Peterson, 2011; Schleier & Peterson, 2010a; Schleier & Peterson, 2010b; Schleier et al., 2012; Schleier, Preftakes & Peterson, 2010) as well as part of regular mosquito abatement program activities, we have noticed that the behavior of the insecticide aerosol cloud may explain some differences in acute mortality of non-target insects that have been observed.

Therefore, given the intrinsic hazard to non-target insects from broad-spectrum ULV insecticides and the equivocal results reported to date and from our observations, we evaluated some factors, such as height, that may influence the ability of an insect to intercept the insecticide at the time of application. To do this, we used the adult convergent lady beetle, *Hippodamia convergens* Guérin-Méneville (Coleoptera: Coccinellidae), a beneficial, predaceous insect (Bjørnson, 2008).

## Materials and Methods

### Test insects

Adult *H. convergens* that were wild-collected from California were purchased from Planet Natural Garden Supply (Bozeman, Montana) immediately before experiments were conducted in 2011 and 2015. This species was chosen because it is one of the more common lady beetles found throughout North America, its use in biological control has become a traditional practice in rural and urban settings, it is easily and inexpensively obtained in large numbers, and adults are hardy and relatively easy to manipulate.

The adults were kept in a terrarium in a 4 °C refrigerator before testing. Equal numbers of males and females were transferred the day of testing to bioassay cages similar to the Clarke cage design described by Fritz et al. (2010): concentric, friction-fit cardboard rings (15.24 cm diameter × 3.81 cm deep) secured by T-310 tulle fabric to each face. Once in the cages, the beetles were kept at room temperature (ca. 23 °C) for approximately 4 h so that there was sufficient time for them to warm up before being taken to the field.

### 2011 study

The field study was performed in August 2011 at or near dusk to coincide with the peak activity period of the target pest, mosquitoes, as recommended by the World Health Organization (WHO, 2003). Permethrin, ([3-phenoxyphenyl]methyl 3-[2,2-dichloroethenyl]-2,2-dimethylcyclopropane carboxylate), was used in a water-based formulation (Aqua-Reslin®) and an oil-based formulation (Permanone® 30-30). Formulations were applied as follows: three replications with Aqua-Reslin at ½ maximum label rate (3.92 g a.i./ha), two replications with Aqua-Reslin at ¼ maximum label rate (1.96 g a.i./ha), two replications with Permanone 30-30 at ½ maximum label rate (3.92 g a.i./ha), and two replications with Permanone 30-30 at ¼ maximum label rate (1.96 g a.i./ha). Therefore, there were nine total applications. Application rates one-quarter and one-half of the maximum label rate of permethrin are commonly used and typically provide satisfactory mortality of adult female mosquitoes (Efird et al., 1991).

Applications of oil- or water-based formulations and application rates were randomly chosen. Replications (see above) were performed over time within the same night (20 min between replications) as well as over different nights. Applications began no earlier than 1900 h between 17 and 30 August 2011.

**Figure 1:**
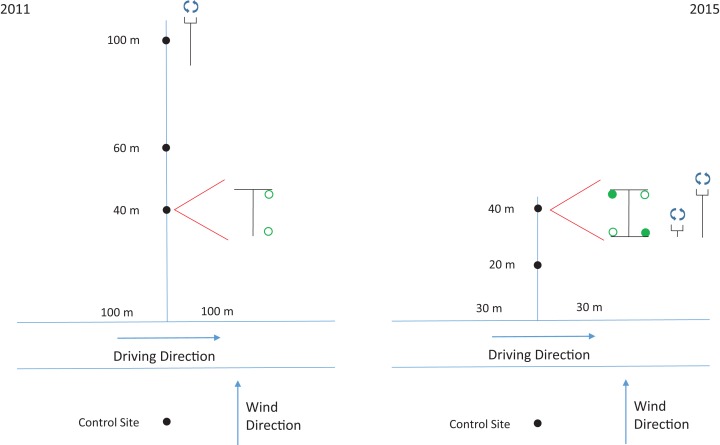
Site layouts for experiments. Site layouts for the 2011 and 2015 experiments. Solid black circles are sample locations. Red lines at 40 m sample location indicate blow-up of sample locations. Each sample location per year would include the same information as that at 40 m. Black “T” or “I” symbols are cage stands (Fig. 3). Solid green circle is cage with front face covered in plastic wrap (Fig. 4). Open green circle is cage with mesh fabric face (Fig. 4). Symbols with blue circular arrows are rotary impingers (Fig. 2).

The field site, located near Bozeman, MT (45°38′47.09″N, 111°24′8.18″W) was a fallow wheat field with very little ground cover. Three T-shaped PVC stands, approximately 1.5-m tall, were placed at 40-, 60-, and 100-m from the spray line (Fig. 1). Each stand had two cages of 25 ± 1 *H. convergens* per cage: one cage suspended from the right arm of the stand 1.5 m above the ground and one cage at ground-level (Fig. 1). A stand was placed 30-m upwind of the point of spray with two control cages positioned the same as the test cages.

All spray applications were made 40-m upwind of the first stand of cages. Air temperature, relative humidity, wind direction, and wind speed were measured during each spray application with a Hobo Micro Station Data Logger (Onset Computer Corporation, Bourne, MA, USA) attached to 12-bit temperature and relative humidity sensors with a solar radiation shield and a wind speed and direction smart sensor positioned 2.5 m above the ground. A rotating droplet impinger (constructed by Cascade County Weed and Mosquito Control, Great Falls, MT, USA) was located 100 m from the spray line and positioned 1.5 m off the ground to sample the aerosol cloud at that location and verify that the droplets moved through the entire study area.

A GUARDIAN® 95 ES (Adapco, Sanford, FL, USA) ULV truck-mounted spray system was used to make all pesticide applications. The spray was initiated and continued for 45 s as the truck drove approximately 16.1 km/hr in a straight 200-m line. After the ULV sprayer was turned off, the aerosol cloud was given 10 min to move through the spray zone. The cages were collected and the *H. convergens* were transferred to 946 ml (1 quart) wide-mouth mason jars to avoid any increased acute mortality from residual permethrin on the cages. Mortality counts were conducted 12 h after the application. Individuals that were unresponsive to being probed or grasped by a pair of forceps were considered dead. Treatment factors were formulation, application rate, distance from spray source (40, 60, and 100 m), and cage height (ground-level and 1.5 m).

### 2015 study

**Figure 2:**
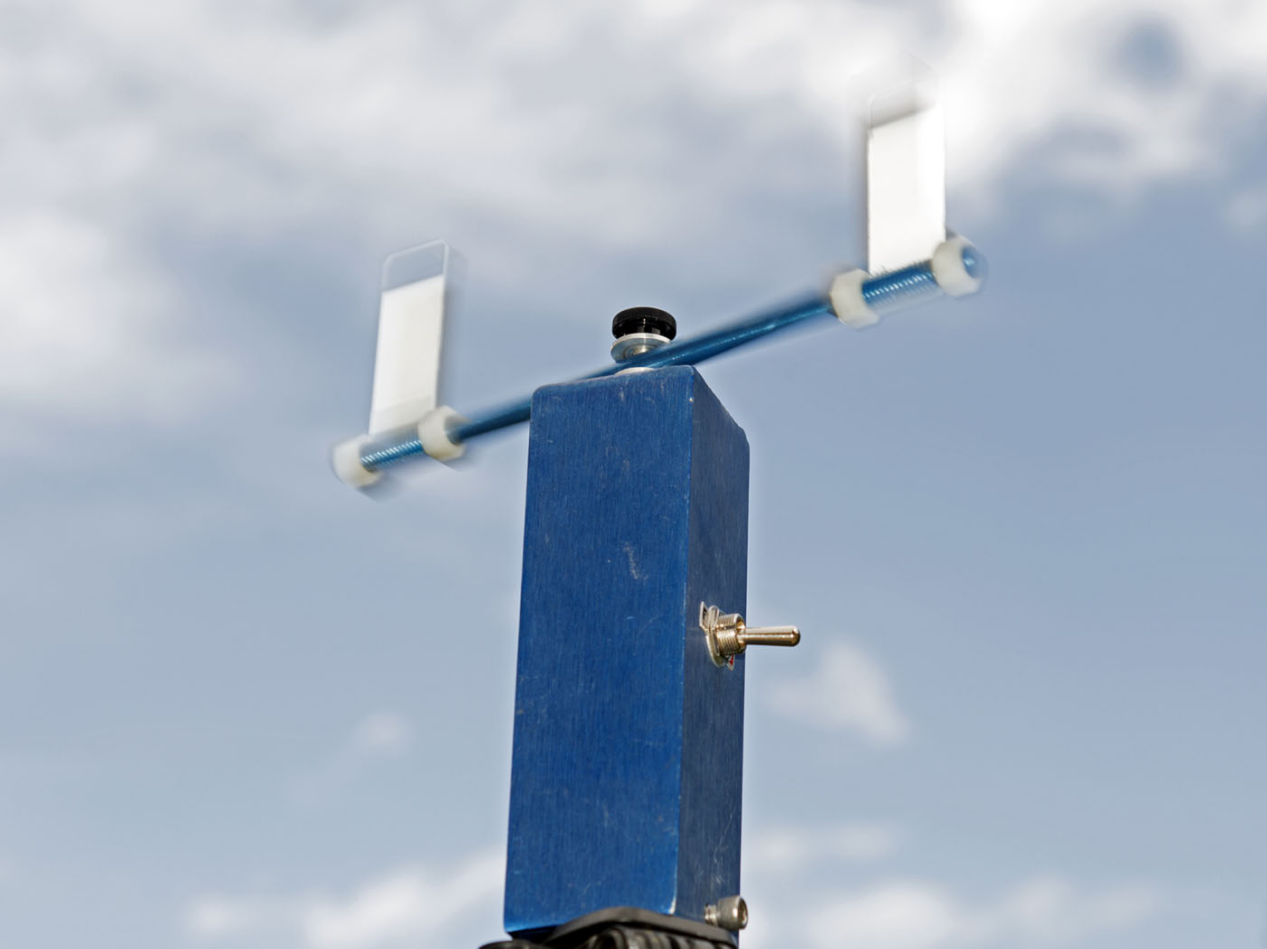
Rotary droplet impinger. Rotary impinger spinning with microscope slides coated with magnesium oxide. The insecticide droplets impinge on the spinning slides, which are then quantified using a microscope and software.

Based on results from the 2011 study, we made some adjustments to the experimental and treatment design for additional research. Although the 2011 study was a simple field bioassay with several treatment replications designed to reveal if there were differences in mortality between individuals at ground-level compared to 1.5 m, it became apparent with the existing design that we could not attribute the height differences in acute mortality to differences in insecticide droplet behavior or the insect’s ability to intercept the droplets. Therefore, we incorporated ground-level and 1.5-m high rotary slide impingers to quantify insecticide droplets (Fig. 2). We also added a covered cage-front treatment factor to better understand the role of objects interfering with the insect’s ability to directly intercept the insecticide droplets.

Methods for the 2015 study were the same as the 2011 study, with the following differences. Field experiments were performed in June and July 2015 at or near dusk. Formulations were applied as follows: five replications with Aqua-Reslin and six replications with Permanone 30-30 at one-half maximum label rate (3.92 g a.i./ha). Therefore, there were 11 total applications. Applications of oil- or water-based formulations were randomly selected. As in 2011, replications (see above) were performed over time within the same night as well as over different nights. Applications began no earlier than 1930 h between 30 June and 9 July 2015.

**Figure 3:**
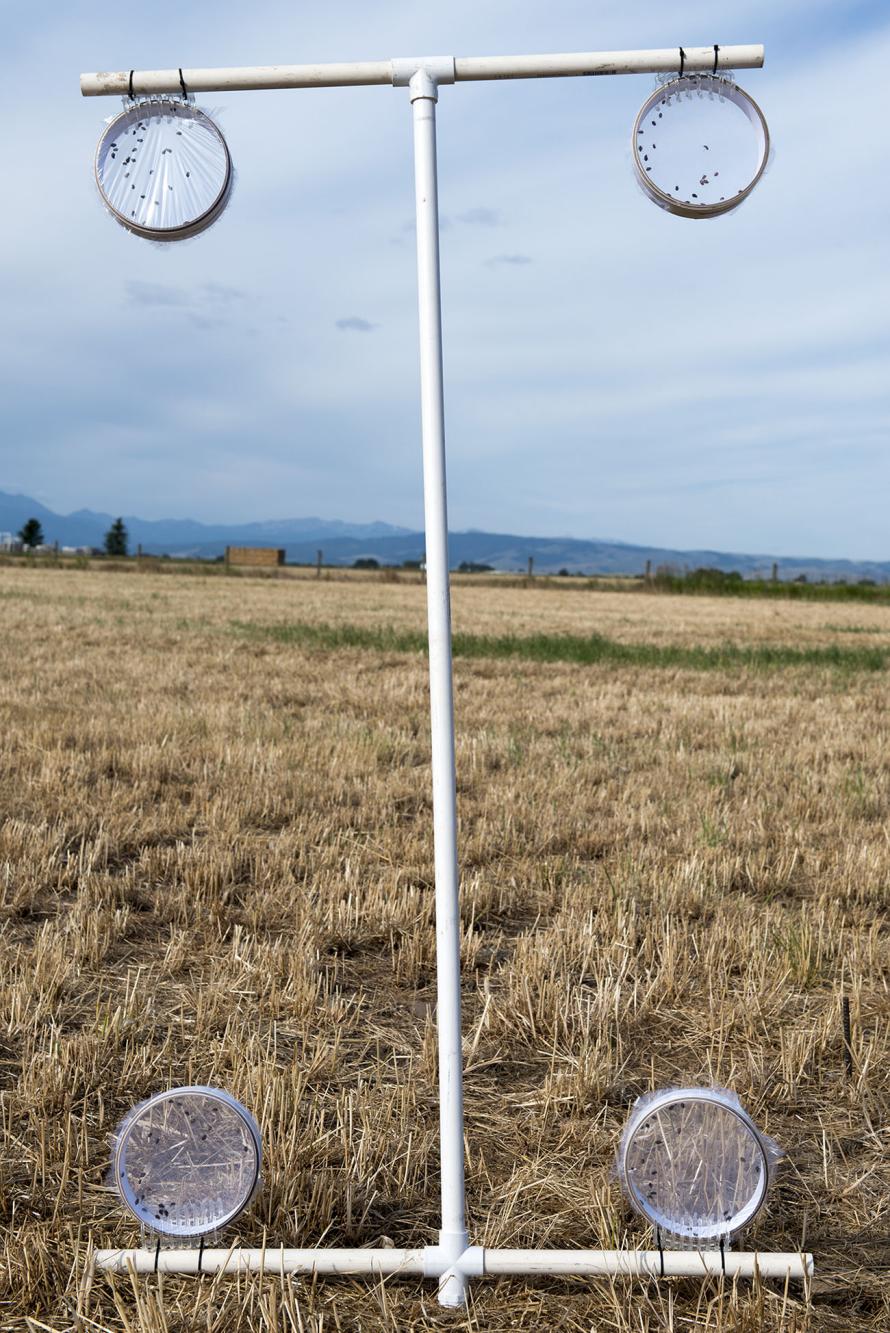
Cage stand with cages. Cage stand with ground-level and 1.5-m high cages.

**Figure 4:**
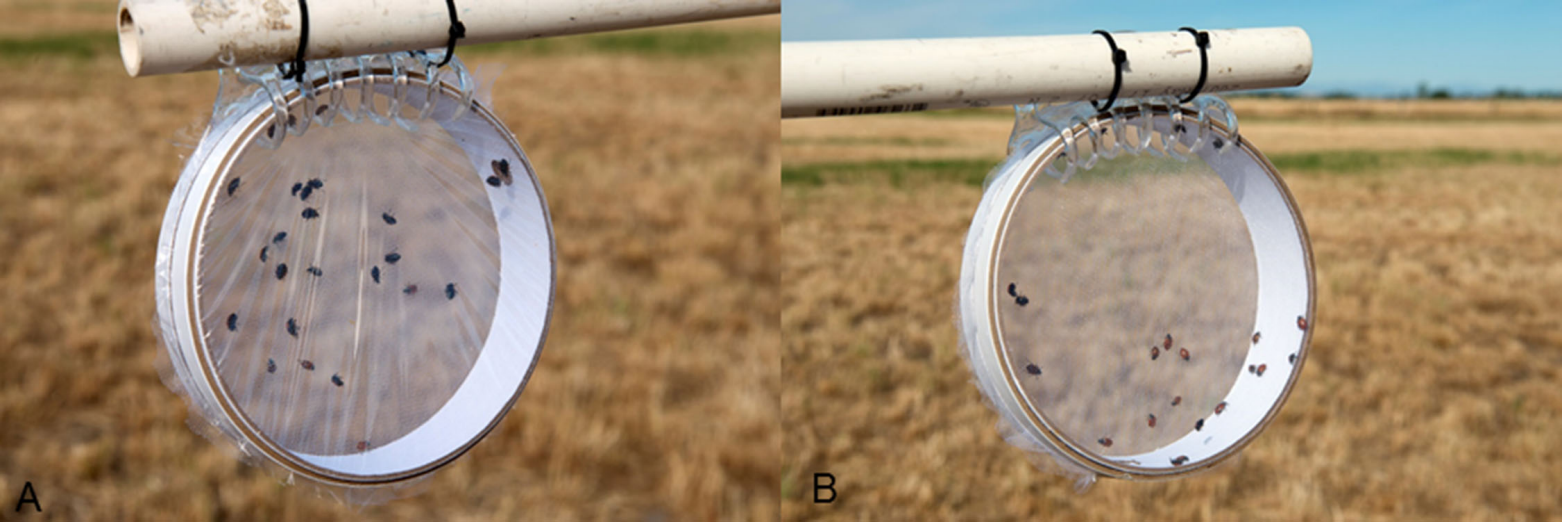
Covered and uncovered cages. Covered cage with plastic polyvinyl chloride wrap over the tulle fabric on the front (A) and uncovered cage with tulle fabric on the front and back (B).

The field site located at the MSU Bozeman Agricultural Research and Teaching Farm in Bozeman, MT (45°39′56.5″N, 111°04′28.6″W) was a recently cut grass hay pasture. The cutting immediately before the study was performed ensured very little ground cover during the experiments, which was similar to the 2011 study. Two I-shaped PVC stands, 1.5 m tall, were placed at 20 and 40 m from the spray line (Fig. 3). Based on 2011 results (see below) and practical reasons, we eliminated the cage stands at 60 and 100 m but added a cage stand at 20 m to determine if beetles closer to the spray source would be more susceptible to acute mortality (Fig. 1). Each stand had four cages of 25 ± 1 *H. convergens* per cage of equal ratios of males and females: two cages suspended from the upper arm of the stand 1.5 m above the ground and two cages suspended from the lower arm at ground-level. All cages were constructed with T-310 tulle fabric secured to each face of the cage. Half the cages (i.e., one each on the upper and lower arms) were covered with plastic polyvinyl chloride wrap over the tulle on the face of the cage that received the spray because of the wind direction (Fig. 4). Placement of the two cage types on each arm of the stand was randomized. A stand was placed 30-m upwind of the point of spray with four control cages positioned the same as the test cages.

Two rotating droplet impingers (Leading Edge Associates, Inc., Fletcher, NC, USA) were located at each stand, one at the height of each cage. Impingers rotated both Teflon- and magnesium oxide-coated slides (for oil- and water-based formulations, respectively) at a speed of 5.6 m/s. Slide dimensions were 75 × 25 mm and were positioned 18.4 cm apart from center to center. The DropVision® measuring system (Leading Edge Associates, Inc., Fletcher, NC, USA) was used to obtain droplet data from slides. This system consists of a specialized compound microscope, with a built-in imaging processor, to analyze droplets on slides while eliminating background objects.

All spray applications were made 20-m upwind of the first stand of cages. The spray was initiated and continued for 20 s as the truck drove 16.1 km/hr in a straight 60-m line. The spray line was perpendicular to the wind direction at each spray. Because there was not a cage stand at 60 or 100 m, the spray line was shorter in 2015 (ASAE, 2009). Air temperature, relative humidity, wind direction, and wind speed were measured during each spray application with a Kestrel 4000 Weather & Environmental Meter (Nielsen-Kellerman Company, Boothwyn, PA, USA). Treatment factors included formulation, distance from spray source (20 and 40 m), cage position (1.5 m or ground-level), and cage type (covered front or uncovered front).

### Data analysis

Acute mortality data were skewed strongly toward either 0 or 100%, depending on whether the individuals were at ground-level or 1.5-m high (2011 and 2015) or in covered or uncovered cages (2015). Therefore, the variability did not meet assumptions of homogeneity using the UNIVARIATE Procedure (SAS 9.3, SAS Institute, Inc., Cary, North Carolina, USA) and the data could not be transformed to meet homogeneity. Therefore, the data could not be statistically analyzed using parametric procedures. Consequently, the data were analyzed within a contingency-table structure using Chi-square and Fisher’s exact test (α = 0.05) procedures (Sokal & Rohlf, 1981) with the FREQ Procedure (SAS 9.3, SAS Institute, Inc., Cary, North Carolina, USA). Logistic regression was used to examine significant effects (α = 0.05) of droplet density measured on the slides, volume median diameter (VMD) measured on the slides, wind speed, temperature, relative humidity with the LOGISTIC Procedure (SAS 9.3, SAS Institute, Inc., Cary, North Carolina, USA). The Wilcoxon rank sign test was used to determine if there were differences in VMD and droplet density between distance and height using R statistical package version 3.2.0 (R Foundation for Statistical Computing, Vienna, Austria) because neither droplet density nor VMD were normally distributed.

In 2011, it was not necessary to correct treatment mortality with control mortality using Abbott’s formula because there was no mortality in the control treatments (Abbott, 1925). In 2015, treatment mortality needed to be corrected for five of the 11 applications. However, control mortality did not exceed 11%.

## Results

### Mortality

#### 2011

The rotary impinger at 100 m collected insecticide droplets during each spray application (mean droplet density = 0.94 ± 0.08 droplets/mm^2^; mean VMD = 15.7 ± 5.73 μm), indicating that the aerosol passed through the 40-, 60-, and 100-m sample distances. There were significant formulation (df = 1; X^2^ = 8.08; P < 0.005) and distance (df = 2; X^2^ = 9.86; P < 0.007) effects on mortality. The acute mortality of *H. convergens* exposed to Aqua-Reslin and Permanone 30-30 was 47.6 and 39.8%, respectively. The mortality of individuals at 40, 60, and 100 m was 47.9, 46.6, and 38.2%, respectively; mortality was significantly lower at 100 m than 40 or 60 m. This is not surprising because 100 m is at the far end of the effective swath width for ground-based ULV applications of insecticides for mosquito control.

**Figure 5:**
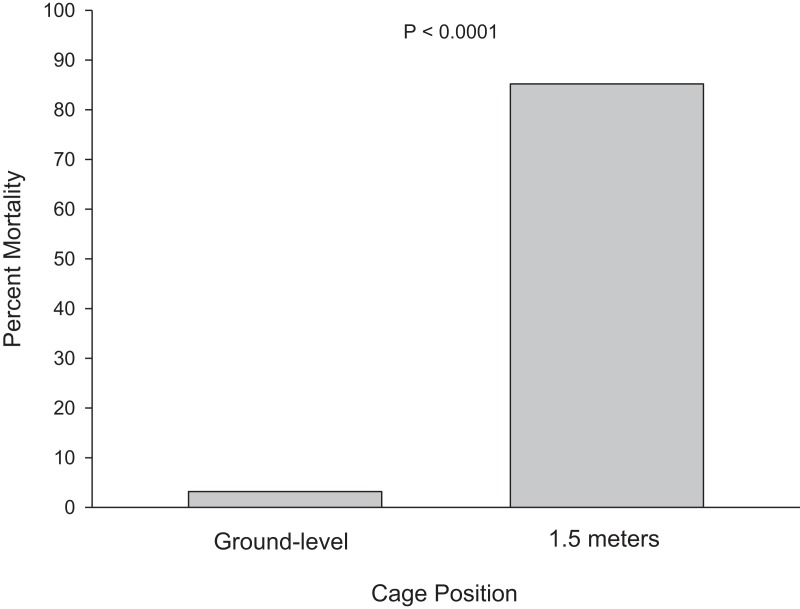
Results from 2011. Cage position was highly significant (P < 0.0001). Error bars are not shown because the data are non-parametric. Mortality was significantly lower at ground level (mean = 3.2%, median = 0%) compared to 1.5 m (mean = 85.2%, median = 100%).

Cage height was highly significant (df = 1; X^2^ = 897.29; P < 0.0001). Acute mortality was significantly lower at ground level (mean = 3.2%, median = 0%) compared to 1.5 m (mean = 85.2%, median = 100%) (Fig. 5). Therefore, mortality was not independent of height. The logistic regression revealed significant effects of wind speed (df = 1; X^2^ = 7.28; P = 0.007) on overall mortality, but the interaction of cage height-by-wind speed was not statistically significant.

#### 2015

Unlike the 2011 study, there were no significant formulation (df = 1; X^2^ = 1.18; P = 0.28) or distance (df = 1; X^2^ = 2.65; P = 0.1) effects on acute mortality. Formulation was not significant possibly because more replications at one rate were conducted in 2015 compared to 2011. Distance was not significant most likely because 20- and 40-m distances were relatively close and we did not have a 100-m distance in 2015.

**Figure 6:**
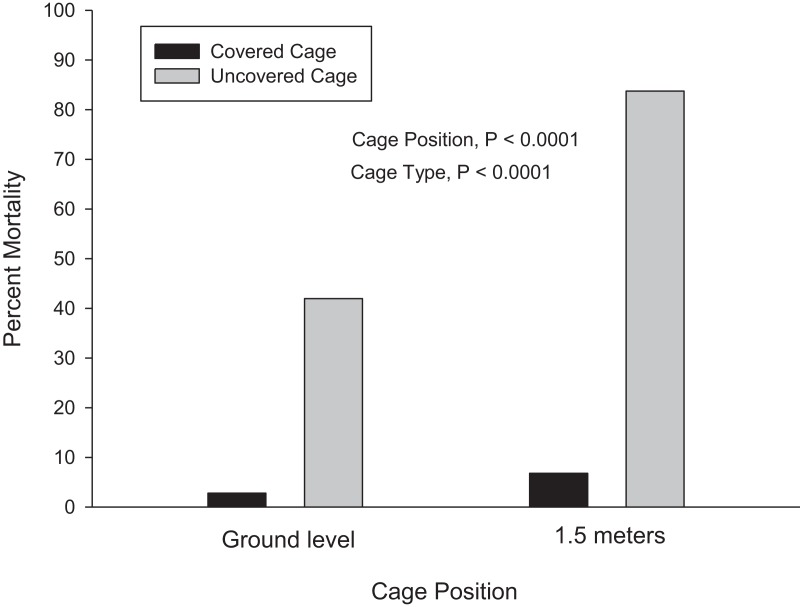
Results from 2015. Cage position and cage type were highly significant (P < 0.0001). Error bars are not shown because the data are non-parametric. Mortality by cage position and cage type was as follows: ground level, covered cage (mean = 2.8%, median = 0.1%); ground level, uncovered cage (mean = 41.9%, median = 9.6%); 1.5 m, covered cage (mean = 6.8%, median = 0%); 1.5 m, uncovered cage (mean = 83.7%, median = 100%).

Like the 2011 study, cage height was highly significant (df = 1; X^2^ = 130.12; P < 0.0001), with acute mortality lower at ground-level compared to 1.5 m (Fig. 6). Cage type was highly significant (df = 1; X^2^ = 840.2; P < 0.0001), with mortality lower in covered cages compared to uncovered cages (Fig. 6). Mortality by cage position and cage type was as follows: ground level, covered cage (mean = 2.8%, median = 0.1%); ground level, uncovered cage (mean = 41.9%, median = 9.6%); 1.5 m, covered cage (mean = 6.8%, median = 0%); 1.5 m, uncovered cage (mean = 83.7%, median = 100%).

The logistic regression revealed significant effects of wind speed (df = 1; X^2^ = 9.18; P = 0.002), relative humidity (df = 1; X^2^ = 7.31; P = 0.007), and temperature (df = 1; X^2^ = 8.79; P = 0.003) on acute mortality in the uncovered cages at ground-level, but not at 1.5 m. This suggests that mortality of individuals in the cages at ground-level was more dependent on these environmental factors than individuals in the cages at 1.5 m.

Surprisingly, there were no significant differences in droplet density and VMD between ground-level and 1.5 m. Therefore, reductions in mortality of individuals at ground-level cannot be explained simply by lower insecticide droplet densities or changes in VMD. There was, however, a significant difference in droplet density between the 20- and 40-m distances (Wilcoxon rank sign test, W = 1,264, P = 0.013) (Table 1).

**Table 1:** Statistical relationships between cage height, distance, VMD, and droplet density. Results from Wilcoxon rank sum test of VMD and droplet density. A P-value < 0.05 indicates that distributions differ between independent variables (Ground-level and 1.5, 20 and 40 m) for each dependent variable (VMD, and Droplets/mm^2^).

Independent variables	VMD		Droplets/mm^2^	
	Test stat	P-value	Test stat	P-value
Ground-level and 1.5 m	1,052	0.4858	808	0.1831
20 and 40 m	1,192	0.0621	1,264	0.0136

#### Weather

The average (± SD) temperature and wind speed during the applications in 2011 were 31.2 ± 1.7 °C and 14.5 ± 4.3 km/hr. The 2011 data for relative humidity could not be used because the sensor was not functioning properly during the applications. The average (± SD) temperature, relative humidity, and wind speed during the applications in 2015 were 26.2 ± 4.2 °C, 38.7 ± 7.6%, and 7.7 ± 3.4 km/hr.

## Discussion

To our knowledge, this is the first study to show that acute mortality of a non-target terrestrial insect when exposed to ULV insecticides for mosquito management is largely determined by aspects of the insect’s position with respect to the insecticide aerosol. Acute mortality was extremely low when the front of the cages (perpendicular to the direction of the insecticide droplets) was covered with plastic wrap and therefore was blocking the insecticide droplets from passing through the cages (Fig. 4), even though droplets were moving past the cages and the back of the cages were not covered with plastic wrap (Table 1). Therefore, the individuals were prevented from directly intercepting the droplets as they moved from the front to the back of the cage and their mortality was much lower than the mortality in the corresponding uncovered cage.

Mortality was also significantly lower for individuals at ground-level compared to 1.5 m (Figs. 5 and 6). This was more pronounced in 2011 compared to 2015. This may be because cages were placed upright on the ground in 2011 compared to 2015 where cages were attached to the lower arm of the cage stand. However, the difference in height was only 6 cm.

By adding rotary slide impingers at ground-level and 1.5 m at approximately the same heights as the cages in 2015, we specifically evaluated if differences in insecticide droplet density and VMD between heights could explain the differences in mortality we observed in 2011. However, droplet density and VMD were not different between ground-level and 1.5 m. This suggests that there may be another factor or factors contributing to lower acute mortality of individuals at ground-level compared with individuals at 1.5 m. As discussed above, the acute mortality of individuals in the cages at ground-level was more dependent on wind speed, relative humidity, and temperature than individuals in the cages at 1.5 m. Therefore, these environmental factors may be interacting with the insecticide droplets in as yet unknown ways to influence mortality.

The results from this study indicate that acute mortality of adult *H. convergens* is significantly determined by both the insect’s ability to directly intercept the insecticide droplets and its height during the spray application. More broadly, our results suggest that mortality to non-target insects may vary considerably based on the ability of the droplets to impinge on the insects because of something blocking the droplets and the height of the insects as the aerosol passes through the area being sprayed. This is not particularly surprising because the technology of using ULV insecticides for mosquito management was developed to target flying adult mosquitoes and to not target insects on foliage and other landscape features (Lofgren, Anthony & Mount, 1973; Mount, 1998; Mount, Biery & Haile, 1996). The insecticides stay aloft and a relatively low percentage of what is applied settles on surfaces such as leaves and soil in the treatment area (Fisher et al., 2015; Hiscox et al., 2006; Preftakes, Schleier & Peterson, 2011; Schleier et al., 2012; Tietze, Hester & Shaffer, 1994).

Our results may provide an explanation for why other studies generally have observed low levels of non-target insect mortality from applications of ULV insecticides for mosquito management. In field bioassays in which cages were placed on the ground, Schleier & Peterson (2010b) did not observe significant differences in mortality of house crickets, *Acheta domesticus* (L.), between control and treated areas for either full-labeled rates of permethrin (Permanone® 10EC) or naled (Trumpet® EC). However, ground-based ULV applications of malathion were shown to have a significant effect on house crickets when caged on the ground, causing 12.5–48.7% acute mortality, depending on their location in residential yards (Tietze et al., 1996). House crickets are more sensitive than some other smaller non-target insects to pyrethroid insecticides (Antwi & Peterson, 2009).

Jensen, Lawler & Dritz (1999) showed that the use of ground-applied ULV permethrin, pyrethrins, and malathion above wetlands had a significant effect on night-flying insects, but abundance recovered 48 h after application. Davis & Peterson (2008) found no effect on terrestrial invertebrates after single and multiple ULV applications of permethrin or δ-phenothrin by ground-based equipment. After airplane-based ULV applications of pyrethrins, Boyce et al. (2007) and Kwan et al. (2009) found no significant effect on large- or medium-bodied insects within the spray zone, but they observed an effect on small-bodied insects such as ants and chironomid midges. Kwan et al. (2009) chose sampling sites to increase the likelihood of detecting effects on non-target insects. These sites were away from significant vegetation canopy and other topographical protective elements in the treatment area that likely provided refugia for insect populations.

After aerial ULV applications of naled in which mosquitoes and *Culicoides* spp. biting midges were greatly reduced in number, Breidenbaugh & de Szalay (2010) observed no statistically significant reductions in numbers or diversities of most common taxa of non-target insects, except for four dipteran families. Breidenbaugh & de Szalay (2010) suggested that the reductions in numbers for the four dipteran families may have been because the insecticide was applied 2 h before sunset and individuals in those families may have directly intercepted the aerosol cloud. Chaskopoulou et al. (2014) did not observe any deleterious effects on honey bees, *Apis mellifera* L., lady beetles, *Cryptolaemus montrouzieri* Mulsant, or green lacewings, *Chrysoperla carnea* (Stephens) after aerial ULV applications of deltamethrin and δ-phenothrin for adult mosquito management.

A few researchers have investigated the sensitivity of butterfly species to aerial applications of ULV mosquito adulticides. Bargar (2012a) and Bargar (2012b) suggested as part of a risk assessment that exposures to the insecticide naled for butterflies in the Florida Keys would exceed levels known to cause mortality. However, the assessment assumed deposition of naled droplets on surfaces would equate to direct exposure of droplets on butterflies. Neither the role of position of the butterfly with respect to the aerosol cloud nor protection from interception of droplets was considered. Similar assumptions and conclusions about butterfly risk have been reported by Zhong et al. (2010), Hoang et al. (2011) and Hoang & Rand (2015).

Studies conducted to date on the effect of mosquito adulticides on non-target insects indicate that there have been equivocal results. Based on our results in the present study, this may be, in part, due to variability in whether the insects directly intercepted the ULV insecticide droplets. Not only may insects close to the ground have reduced mortality, but insects on plants and other structures in the landscape not directly intercepting the aerosol cloud, such as those behind rocks, leaves, and bark, may be unaffected or experience low mortality.

This is further supported by several studies with honey bees. These studies suggest that when the bees are inside of the hive boxes at the time of the insecticide application, as is typical, there can be low mortality. Caron (1979) exposed caged honey bees and hives to ground applications of ULV malathion, naled, and pyrethrum. Caged bees experienced significant mortality for all treatments, but the mortality decreased rapidly with increasing distance from the point of application. Exposure to hives revealed that night applications had no effect on colonies, whereas day applications of malathion caused significant mortality as indicated by dead bee traps. Hester et al. (2001) observed significant bee mortality within 15 m of the spray source in hives that were exposed to four ground applications of ULV malathion both in open fields and in a forested environment. There was no effect on hive weight or health of the colony over a season. Zhong et al. (2004) and Zhong et al. (2003) observed similar results with aerially applied naled and noted that bees clustering outside of the hive (termed “bearding”) were most susceptible.

Future research is needed that involves additional experimental manipulation of droplet interception by insects. We placed plastic wrap immediately in front of the tulle fabric at the front face of the cage. A subsequent assessment could involve placing plastic sheets at increasing distances from the front of cages to determine their effect on acute mortality. This would provide more information on the role of objects interfering with the insect’s ability to directly intercept the insecticide droplets. Because droplet density and VMD did not explain differences in mortality between individuals in ground-level cages compared to individuals at 1.5 m, additional research is needed that characterizes the environmental factors that affect mortality of insects at ground-level. In addition, because we and other researchers have only characterized acute mortality, sublethal effects could be considered in future research.

Our results demonstrate the importance of considerations of exposure that go well beyond deposition of ULV insecticides on surfaces and extrapolation of that deposition to risk to non-target insects. Insecticides currently used as outdoor space sprays for adult mosquito management are broad-spectrum and highly toxic to many insects. However, ULV technology most likely mitigates exposures to most insects because of small droplet sizes, extremely low application rates, and application timing. Therefore, the exposure component of risk is very important to understand when assessing risk to non-target insects and other arthropods.

## Supplemental Information

10.7717/peerj.2167/supp-1 Supplemental Information 1 2011 Data.

10.7717/peerj.2167/supp-2 Supplemental Information 2 2015 Data.

## Additional Information and Declarations

### Data Deposition

The following information was supplied regarding data availability: The raw data has been supplied as Supplemental Dataset Files.
